# Supplementary material for: A rapid detection tool for VT isolates of Citrus tristeza virus by immunocapture-reverse transcriptase loop-mediated isothermal amplification assay
Source: PLoS One. 2019 Sep 5;14(9):e0222170. doi: 10.1371/journal.pone.0222170 (PMC6728045; doi:10.1371/journal.pone.0222170)
Supplement: S1 Table — (DOCX) [file pone.0222170.s001.docx]

**S1 Table.** **RT-qPCR primers and probes used in a matrix to genotype *Citrus tristeza virus* isolates in this study.**

| **Strain** | **Primer/Probe** | **Name** | **sequence** | **Reference** |
| --- | --- | --- | --- | --- |
| CTV - Coat  Protein universal | Forward Primer | P25F | AGCRGTTAAGAGTTCATCATTRC | [23] |
|  | Reverse Primer | P25R | TCGRTCCAAAGTTTGTCAGACA |  |
|  | Probe | CP probe | CRCCACGGGYATAACGTACACTCGG |  |
| VT | Forward Primer | VT F | GCTGCGGGAATYGGTGTA | This study |
|  | Reverse Primer | VT R | CGAAAGTCGAGGACYTGAAG |  |
|  | Probe | VT P | 6FAM/ CAAATTGCCCACTACGCCCATAC |  |
| T30 | Forward Primer | VT/T30/T68-RT-F | CGATGGTCAAGCGGACGACTT | [4] |
|  | Reverse Primer | Common RT Reverse | GCAAACATCTCGACTCAACTACC |  |
|  | Probe | T30 | TGAACAAACGATCAACCAGTCATC |  |
| T36 | Forward Primer | P27F | TACGYGATTTGGGWAAGTAYT | [23] |
|  | Reverse Primer | P27R | GACCCTTAAAGCAGTGCTCA |  |
|  | Probe | T36 | ACGGTAACATTATACTATCCC |  |
| S1 | Forward Primer | S1F | CGTTGCGCGCTAAGTTT | [20] |
|  | Reverse Primer | S1R | GACACTCCAGCTTCGTCTTA |  |
|  | Probe | S1 | TCGTCACCGTCTGGGAGATTGTCT |  |
| RB/S1 | Forward Primer | P27F | TACGYGATTTGGGWAAGTAYT | [23] |
|  | Reverse Primer | P27R | GACCCTTAAAGCAGTGCTCA |  |
|  | Probe | T36NS | CGGTARTATYATRCCATCCT |  |
| RB/T36 | Forward Primer | RB/T36F | CACAACAGGGATCCGGAATAG |  |
|  | Reverse Primer | RB/T36R | CCAGAAGCTTCCTCTGAGTTT | This study |
|  | Probe | RB/T36 probe (antisense) | TGTGGGAATATTAGAGCTTAAAGGCTGGAC |  |
