# Supplementary material for: A rapid detection tool for VT isolates of Citrus tristeza virus by immunocapture-reverse transcriptase loop-mediated isothermal amplification assay
Source: PLoS One. 2019 Sep 5;14(9):e0222170. doi: 10.1371/journal.pone.0222170 (PMC6728045; doi:10.1371/journal.pone.0222170)
Supplement: S2 Table — (DOCX) [file pone.0222170.s002.docx]

**S2 Table.** **Temperature gradient of reverse transcriptase LAMP assay for detection of CA-VT-AT39 strain of *Citrus tristeza virus***

|  | **Time of amplification (min:sec) (Melting temperature ºC)** | | | | |
| --- | --- | --- | --- | --- | --- |
| **ºC/Replication (R)** | **R1** | **R2** | **R3** | **mean** | **SD** |
| 59 | 11:15 (88.4) | 11:00 (88.4) | 10:45 (87.9) | 11:00 (88.2) | 0.0104 |
| 60 | 10:45 (88.5) | 10:30 (88.5) | 10:30 (88.1) | 10:35 (88.4) | 0.0060 |
| 61 | 9:45 (88.3) | 9:45 (88.7) | 9:30 (88.3) | 9:40 (88.4) | 0.0060 |
| 62 | 9:15 (88.7) | 9:00 (88.7) | 8:45 (88.2) | 9:00 (88.5) | 0.0104 |
| 63 | 8:45 (88.6) | 8:30 (88.6) | 8:30 (88.2) | 8:35 (88.5) | 0.0060 |
| 64 | 8:30 (88.7) | 8:15 (88.7) | 8:15 (87.8) | 8:20 (88.4) | 0.0060 |
| **65** | **8:30 (88.2)** | **8:00 (88.6)** | **8:15 (87.8)** | **8:15 (88.2)** | **0.0104** |
| 66 | 8:45 (88.6) | 8:15 (88.6) | 8:15 (88.2) | 8:25 (88.5) | 0.0120 |
