# Supplementary material for: A rapid detection tool for VT isolates of Citrus tristeza virus by immunocapture-reverse transcriptase loop-mediated isothermal amplification assay
Source: PLoS One. 2019 Sep 5;14(9):e0222170. doi: 10.1371/journal.pone.0222170 (PMC6728045; doi:10.1371/journal.pone.0222170)
Supplement: S3 Table — (DOCX) [file pone.0222170.s003.docx]

**S3 Table.** **Detection of** **CA-VT-AT39 RNA by RT-ddPCR and RT-LAMP assays**

| **RNA dilution** | **ddPCR** | | | | | | **RT-LAMP** | | | | |
| --- | --- | --- | --- | --- | --- | --- | --- | --- | --- | --- | --- |
|  | **Measured copies per 20 µl well** | | | | **Poisson SEM** | **Total SEM** | **Time of amplification (min:sec)** | | | | **SD** |
|  | **R1** | **R2** | **R3** | **Mean** |  |  | **R1** | **R2** | **R3** | **Mean** |  |
| 20 ng | NA | NA | NA | NA | NA | NA | 6:30 | 6:15 | 6:30 | 6:25 | 0.006 |
| 2 ng | 14100.0 | 14020.0 | 13900.0 | 14020.0 | 5.2 | 5.2 | 7:15 | 7:00 | 7:15 | 7:10 | 0.006 |
| 0.2 ng | 1712.0 | 1698.0 | 1656.0 | 1688.0 | 1.6 | 1.6 | 8:15 | 8:00 | 8:15 | 8:10 | 0.006 |
| 0.02 ng | 172.0 | 178.0 | 176.0 | 176.0 | 0.5 | 0.5 | 9:15 | 9:00 | 9:15 | 9:10 | 0.006 |
| 0.002 ng | 8.4 | 16.8 | 9.0 | 11.4 | 0.1 | 0.2 | 10:00 | 10:15 | 11:30 | 10:35 | 0.033 |
| 0.0002 ng | 1.2 | 3.8 | 5.4 | 3.4 | 0.1 | 0.1 | NA | NA | NA | NA | NA |
| Healthy | 0 | 0 | 0 | 0 | 0 | 0 | 0 | 0 | 0 | 0 | 0 |
| NTC | 0 | 0 | 0 | 0 | 0 | 0 | 0 | 0 | 0 | 0 | 0 |
