# Supplementary material for: A rapid detection tool for VT isolates of Citrus tristeza virus by immunocapture-reverse transcriptase loop-mediated isothermal amplification assay
Source: PLoS One. 2019 Sep 5;14(9):e0222170. doi: 10.1371/journal.pone.0222170 (PMC6728045; doi:10.1371/journal.pone.0222170)
Supplement: S4 Table — (DOCX) [file pone.0222170.s004.docx]

**S4 Table.** **Optimization of *Citrus tristeza virus* (CTV) polyclonal antibody concentration for Immuno-capture RT-LAMP assay for VT-CTV detection**

| **Antibody concentration** | **Time of amplification (min:sec)** | | | | **SD** |
| --- | --- | --- | --- | --- | --- |
|  | **R1** | **R2** | **R3** | **Mean** |  |
| 1:500 | 6:45 | 6:45 | 6:45 | 6:45 | 0.000 |
| 1:1000 | 6:45 | 6:45 | 6:45 | 6:45 | 0.000 |
| 1:2000 | 7:15 | 7:00 | 7:00 | 7:05 | 0.006 |
| 1:4000 | 8:15 | 8:15 | 8:15 | 8:15 | 0.000 |
| 1:8000 | 8:45 | 8:45 | 8:45 | 8:45 | 0.000 |
| 1:16000 | 9:00 | 8:45 | 9:15 | 9:00 | 0.010 |
| Healthy | 0 | 0 | 0 | 0 | 0 |
