# Supplementary material for: A rapid detection tool for VT isolates of Citrus tristeza virus by immunocapture-reverse transcriptase loop-mediated isothermal amplification assay
Source: PLoS One. 2019 Sep 5;14(9):e0222170. doi: 10.1371/journal.pone.0222170 (PMC6728045; doi:10.1371/journal.pone.0222170)
Supplement: S5 Table — (DOCX) [file pone.0222170.s005.docx]

**S5 Table.** **Dilution of citrus leaf crude extract by IC-RT-LAMP assay for detection of VT strains of *Citrus tristeza virus***

| **Sample concentration** | **Time of amplification (min:sec)** | | | | **SD** |
| --- | --- | --- | --- | --- | --- |
|  | **R1** | **R2** | **R3** | **Mean** |  |
| 1:500 | 5:09 | 5:09 | 5:18 | 5:12 | 0.08 |
| 1:1000 | 6:00 | 6:00 | 6:00 | 6:00 | 0 |
| 1:2000 | 6:27 | 6:27 | 7:00 | 6:38 | 0.31 |
| 1:4000 | 7:27 | 7:27 | 7:27 | 7:27 | 0 |
| 1:8000 | 8:27 | 8:27 | 8:27 | 8:27 | 0 |
| 1:16000 | 10:00 | 9:27 | 10:00 | 9:49 | 0.31 |
| Healthy | 0 | 0 | 0 | 0 | 0 |
| NTC | 0 | 0 | 0 | 0 | 0 |
