# Supplementary material for: A rapid detection tool for VT isolates of Citrus tristeza virus by immunocapture-reverse transcriptase loop-mediated isothermal amplification assay
Source: PLoS One. 2019 Sep 5;14(9):e0222170. doi: 10.1371/journal.pone.0222170 (PMC6728045; doi:10.1371/journal.pone.0222170)
Supplement: S6 Table — aTRS: Township/Range/Section is basic unit in the US Public Land Survey System and it is a square piece of land one mile by one mile containing 640 acres. http://www.jsu.edu/dept/geography/mhill/phygeogone/trprac.html. bCTV strain differentiation was performed with four-sets of duplex RT-qPCR tests with specific primer pair and hydrolysis probes as follows: 1) CP-CY5 and VT (new)-VIC; 2) RB/T36-VIC and T36-FAM; 3) S1-FAM and RB/S1-TET; 4) VT3-FAM and T30-VIC (data in bold). cNew VT primer/probe validated by side-by side comparison with the VT3 primer/probe (data in bold). dNA = No Reaction. eThis T/R/S locale has no section designation. (DOCX) [file pone.0222170.s006.docx]

**S6 Table. Validation of new primer and hydrolysis probe for VT genotype strains and mixtures of 43 California field isolates of *Citrus tristeza virus* (CTV) isolates by Reverse Transcription-quantitative Polymerase Chain Reaction Assay (RT-qPCR) in comparison with a matrix of other genotype-specific primer/probes.**

| **Sample ID** |  | Collection locale  (County) | RT-qPCR Cycle thresholds (Cq) for primers/probes to differentiate CTV genotypes^b^ | | | | | | | | **Genotype** |
| --- | --- | --- | --- | --- | --- | --- | --- | --- | --- | --- | --- |
|  | **TRS^a^** |  | **CP-CY5** | **VT (new)^c^** | **VT3** | **RB/S1** | **RB/T36** | **T36** | **S1** | **T30** |  |
| CCTEA 00452 | 14/24/33 | Fresno | 16.6 | **22.3** | **19.5** | NA^d^ | NA | NA | NA | NA | VT |
| P108 B-B (CCTEA 00450) | 14/24/33 | Fresno | 16.5 | **23.8** | **20.5** | NA | NA | NA | NA | NA | VT |
| P109-AT78 (CCTEA 00451) | 14/24/33 | Fresno | 17.3 | **24.0** | **21.1** | NA | NA | NA | NA | NA | VT |
| P108 AT39 | 14/24/33 | Fresno | 15.4 | **22.3** | **19.9** | NA | NA | NA | 18.3 | NA | VT/S1 |
| CCTEA 14710 | 15/24/33 | Fresno | 18.5 | **24.3** | **20.7** | 18.5 | 20.1 | NA | 27.4 | NA | VT/RB/S1 |
| CCTEA 99416 | 15/24/25 | Fresno | 19.9 | **24.7** | **21.2** | 17.9 | 20.7 | NA | 27.1 | NA | VT/RB/S1 |
| P108A | 14/24/33 | Fresno | 17.9 | **25.5** | **21.2** | 17.6 | 20.3 | NA | 20.7 | 24.8 | VT/RB/S1/T30 |
| P108 AT35 | 14/24/33 | Fresno | 21.4 | **NA** | **NA** | 18.3 | 18.9 | NA | NA | 24.6 | T30/RB |
| P1 | 13/23/24 | Fresno | 19.3 | **NA** | **NA** | NA | NA | NA | NA | 25.4 | T30 |
| CCTEA 107 | 18/26/13 | Tulare | 17.9 | **22.7** | **21.3** | NA | NA | NA | NA | NA | VT |
| RH | 18/26/13 | Tulare | 18.0 | **24.9** | **19.5** | NA | NA | NA | NA | NA | VT |
| JG348 | 18/27/18 | Tulare | 19.7 | **25.5** | **22.5** | NA | NA | NA | NA | NA | VT |
| Sevil 1 | 17/25/14 | Tulare | 17.7 | **24.7** | **21.3** | NA | NA | NA | NA | NA | VT |
| CCTEA 96014 | 20/26/11 | Tulare | 20.6 | **25.2** | **24.0** | 19.0 | 21.1 | NA | NA | NA | VT/RB |
| CCTEA 737 | 18/26/09 | Tulare | 18.5 | **24.7** | **22.6** | 18.7 | 22.1 | NA | NA | NA | VT/RB |
| CCTEA 737 | 18/26/09 | Tulare | 20.4 | **25.2** | **23.3** | 19.6 | 23.3 | NA | 20.9 | 28.3 | VT/RB/S1/T30 |
| P LREC 51 | 18/27/16 | Tulare | 18.9 | **NA** | **NA** | NA | NA | NA | NA | 24.0 | T30 |
| P McE-2 | 18/26/26 | Tulare | 17.2 | **NA** | **NA** | NA | NA | NA | NA | 22.8 | T30 |
| CCTEA 115 | 20/27/09 | Tulare | 22.5 | **NA** | **NA** | 18.8 | 19.1 | NA | NA | NA | RB |
| CCTEA 15 | 18/26/33 | Tulare | 21.7 | **NA** | **NA** | 17.7 | 17.6 | NA | NA | NA | RB |
| CCTEA 109 | 20/27/18 | Tulare | 21.8 | **NA** | **NA** | 18.5 | NA | NA | NA | NA | RB |
| CCTEA 99395 | 18/27/16 | Tulare | 17.6 | **NA** | **NA** | 18.0 | NA | NA | NA | 22.3 | T30/RB |
| CCTEA 99423 | 20/27/22 | Tulare | 19.0 | **NA** | **NA** | 19.0 | NA | NA | NA | 23.1 | T30/RB |
| P54 (CCTEA 65) | 18/2720 | Tulare | 22.3 | **NA** | **NA** | 17.7 | NA | NA | 18.4 | NA | S1 |
| CCTEA 07527 | 18/27/16 | Tulare | 21.1 | **NA** | **NA** | 18.0 | NA | NA | 18.0 | NA | S1 |
| CCTEA 10629 | 18/27/16 | Tulare | 21.9 | **NA** | **NA** | 19.2 | NA | NA | 18.7 | NA | S1 |
| P-S1 Citron 668 | 18/27/16 | Tulare | 21.8 | **NA** | **NA** | 18.9 | NA | NA | 18.7 | NA | S1 |
| CCTEA 142 | 26/26/23 | Tulare | 21.6 | **NA** | **NA** | 21.5 | 25.0 | NA | 21.6 | 28.3 | RB/S1/T30 |
| CCTEA 11661 | 22/27/14 | Tulare | 19.0 | **NA** | **NA** | NA | 20.1 | 19.5 | NA | NA | T36 |
| CCTEA 208 | 30/29/02 | Kern | 16.8 | **22.4** | **20.8** | NA | NA | NA | NA | NA | VT |
| CCTEA 280 | 30/29/02 | Kern | 17.3 | **21.6** | **21.1** | NA | NA | NA | NA | NA | VT |
| CCTEA 96271 | 25/26/36 | Kern | 20.4 | **NA** | **NA** | NA | 34.1 | NA | NA | 24.5 | T30 |
| P25 | 26/26/23 | Kern | 19.9 | **NA** | **NA** | NA | NA | NA | NA | 24.3 | T30 |
| P81-5A | 26/26/23 | Kern | 19.5 | **NA** | **NA** | NA | NA | NA | NA | 24.8 | T30 |
| CCTEA 206 | 30/29/02 | Kern | 21.5 | **NA** | **NA** | 20.9 | 23.8 | NA | 20.2 | 26.8 | RB/S1/T30 |
| P100-2D | 2/20/00^e^ | Ventura | 18.2 | **NA** | **NA** | NA | NA | NA | NA | 23.4 | T30 |
| P Filmr-1 | 4/19/31 | Ventura | 18.8 | **NA** | **NA** | 18.2 | 19.9 | NA | NA | 24.1 | T30/RB |
| P 702 Filmr AT 25a | 4/19/31 | Ventura | 19.8 | **NA** | **NA** | 18.7 | 19.1 | NA | NA | 24.9 | T30/RB |
| P-568-2 | Unknown | Riverside | 14.0 | **21.1** | **16.0** | NA | NA | NA | NA | NA | VT |
| P-568-11 | Unknown | Riverside | 14.2 | **21.4** | **16.4** | NA | NA | NA | NA | 24.9 | VT/T30 |
| CCTEA 558 | Unknown | Riverside | 16.8 | **24.0** | **18.3** | 16.6 | 18.6 | NA | NA | 23.0 | VT/RB/T30 |
| SY553 Meyer lemon | Unknown | Riverside | 20.6 | **27.0** | **22.7** | 19.5 | 22.8 | NA | 27.6 | NA | VT/RB/S1 |
| CCTEA 519 | Unknown | Riverside | 20.8 | **NA** | **NA** | 17.6 | NA | NA | 17.7 | NA | S1 |

^a^TRS: Township/Range/Section is basic unit in the US Public Land Survey System and it is a square piece of land one mile by one mile containing 640 acres. <http://www.jsu.edu/dept/geography/mhill/phygeogone/trprac.html>

^b^CTV strain differentiation was performed with four-sets of duplex RT-qPCR tests with specific primer pair and hydrolysis probes as follows: 1) CP-CY5 and VT (new)-VIC; 2) RB/T36-VIC and T36-FAM; 3) S1-FAM and RB/S1-TET; 4) VT3-FAM and T30-VIC (data in bold).

^c^New VT primer/probe validated by side-by side comparison with the VT3 primer/probe (data in bold).

^d^NA = No Reaction

^e^This T/R/S locale has no section designation.
