# Supplementary material for: A rapid detection tool for VT isolates of Citrus tristeza virus by immunocapture-reverse transcriptase loop-mediated isothermal amplification assay
Source: PLoS One. 2019 Sep 5;14(9):e0222170. doi: 10.1371/journal.pone.0222170 (PMC6728045; doi:10.1371/journal.pone.0222170)
Supplement: Attachment — Submitted filename: Response to reviewer(s) PONE D-19-14855.docx [file pone.0222170.s007.docx]

Corrected PONE-D-19-14855

**Title:** A rapid detection tool for VT isolates of *Citrus tristeza virus* by immunocapture-reverse transcriptase loop-mediated isothermal amplification assay

1. When submitting your revision, we need you to address these additional requirements.

Please ensure that your manuscript meets PLOS ONE's style requirements, including those for file naming. The PLOS ONE style templates can be found at

http://www.journals.plos.org/plosone/s/file?id=wjVg/PLOSOne_formatting_sample_main_body.pdf and http://www.journals.plos.org/plosone/s/file?id=ba62/PLOSOne_formatting_sample_title_authors_affiliations.pdf

**Response:** Corrected

2. We noticed you have some minor occurrence of overlapping text with the following previous publication(s), which needs to be addressed:

https://apsjournals.apsnet.org/doi/10.1094/PHYTO-100-4-0319

https://www.nature.com/articles/s41598-017-13881-4

In your revision ensure you cite all your sources (including your own works), and quote or rephrase any duplicated text outside the methods section. Further consideration is dependent on these concerns being addressed."

**Response:** Corrected see Ln 60-61; Ln 109-110

3. In your Methods section, please provide additional location information, including geographic coordinates for the data set if available.

**Response:** See ln 117-118 and Township/Range/Section locale for each plot were added to Table 4 and each isolate in S6 Table.

4.We note that you have stated that you will provide repository information for your data at acceptance. Should your manuscript be accepted for publication, we will hold it until you provide the relevant accession numbers or DOIs necessary to access your data. If you wish to make changes to your Data Availability statement, please describe these changes in your cover letter and we will update your Data Availability statement to reflect the information you provide.

**Response:** We will change our Data Availability statement since I do not think this is necessary. If it is a requirement, we will gladly do so.

Additional Editor Comments (if provided):

Please could you address the following comments before your manuscript is sent out for external review:

*************************************************

Generalities: The title mentions the VT strain, yet there are absolutely no references to VT genomic uniqueness especially in its 5'end as demonstrated already in 1996 following its complete sequencing (Mawassi et al 1996) and comparison with T36 (Karasev et al, 1995).

**Response:**

- Sequence comparison of CTV-VT and CTV-T36 showed that the 5UTR and ORF1 of VT had only 60% identity and 60-70% respectively (Mawassi et al. 1996).
- In general, the CTV are more conserved towards 3’region and variable towards the 5’ region. But the 5`UTR region is conserved within the genotypes, that’s the reason we selected the 5’UTR-ORF 1a region for designing VT specific primers and probes.

- The alignment between the VT isolates from Florida and California with the other CTV genotypes reported elsewhere. The yellow highlight shows the location of VT-TaqMan Probe specific to VT genotypes located within 5`UTR. The nucleotide sequence conservation among the VT isolates in 5`UTR can be seen in the above screen shot obtained from MEGA-X software.
- G. Ananthakrishnan et al. 2010, J. Virological methods. Developed multiplex RT-qPCR to detect VT genotype using probes specific to 5’UTR, ORF 1a and VT ORF 2. In fig 4, they had showed that 5’UTR of VT strain showed less CT value (more sensitive), compared to probes specific to ORF1a and ORF2.

Similarly, nothing is mentioned on VT: unique biological characteristics, subdivision into different isolates reported which cause r both quick decline QD and stem pitting (SP), while others cause SP of grapefruits with minimal or no effects on orange trees grafted on sour oranges.

**Response:** A better description of VT strains and their diversity is added Ln 77-84.

On the specifics, the authors did mention previous reports on CTV genotyping by Northern Hybridization and sequential analyses of enzyme immunoassays and capillary electrophoresis-single-strand conformation polymorphisms. J Virol Methods. 181:139-47. Furthermore, in the above JVM paper tests were conducted on RNA recovered from positively reacting ELISA wells, thus directing genotyping only for the positively reacting citrus trees.

**Response:** The main aim of this manuscript is to develop field-oriented detection technique to detect VT strains of CTV to use for disease management. The IC-RT-LAMP assay developed in this study can be used by farmers/unskilled personal to detect if VT strains in the field without need to extract and purify RNA from the sample. This manuscript is not focused on genotyping of CTV strains. Rather, we used TaqMan probes to validate the sensitivity and exclusivity of the IC-RT-LAMP assay.

A previous report on RT-LAMP assay for sensitive detection of CTV by Warghane in J Virol Methods. 2017 Dec;250:6-10. doi: 10.1016/j.jviromet.2017.09.018. Epub 2017 Sep 21 is also missing.

**Response:** Page 4, Line 69-70. “Warghane et al [13] showed the sensitivity of RT-LAMP is 100 times more than RT-PCR using the RNA extracted from the CTV infected leaf samples” was mentioned in introduction.

******************************************************

[Note: HTML markup is below. Please do not edit.]

Reviewers' comments:

[NOTE: If reviewer comments were submitted as an attachment file, they will be attached to this email and accessible via the submission site. Please log into your account, locate the manuscript record, and check for the action link "View Attachments". If this link does not appear, there are no attachment files to be viewed.]

While revising your submission, please upload your figure files to the Preflight Analysis and Conversion Engine (PACE) digital diagnostic tool, https://pacev2.apexcovantage.com/. PACE helps ensure that figures meet PLOS requirements. To use PACE, you must first register as a user. Registration is free. Then, login and navigate to the UPLOAD tab, where you will find detailed instructions on how to use the tool. If you encounter any issues or have any questions when using PACE, please email us at figures@plos.org. Please note that Supporting Information files do not need this step.
