# Supplementary material for: A rapid detection tool for VT isolates of Citrus tristeza virus by immunocapture-reverse transcriptase loop-mediated isothermal amplification assay
Source: PLoS One. 2019 Sep 5;14(9):e0222170. doi: 10.1371/journal.pone.0222170 (PMC6728045; doi:10.1371/journal.pone.0222170)
Supplement: Attachment — Submitted filename: Response to reviewer(s) PONE D-19-14855 ver4.docx [file pone.0222170.s008.docx]

Corrected PONE-D-19-14855.ver4

**Title:** A rapid detection tool for VT isolates of *Citrus tristeza virus* by immunocapture-reverse transcriptase loop-mediated isothermal amplification assay

Additional Editor Comments (if provided):

The manuscript has too many language errors to be accepted as is. Here is a list of necessary changes. A proof reader should be asked to deal with other possible problems. I made all requested edits below and had my new postdoc read and review grammar and style. She found many more errors which were corrected. She also noted inconsistencies in reporting amplification times sometimes using sec:min; other times using minutes and tens of minutes. In Ver. 4, all times are reported as min:sec. S2 Table edited to label Melting temperature °C

l. . 116 "leaf samples" corrected

l.121 number disagreement dara are corrected
l.121 Table S5 corrected
l. 124-5 temperatures corrected
l. 164 were used as negative corrected
L. 238 "extracts was grounded ???? were ground corrected
l. 245 used in further. corrected
l. 252 times were corrected
l.256 at different ratios corrected

New comments on second review:
The language usage still requires considerable work before the manuscript is acceptable. Research is fine. Language usage revised as appropriate
l. 92 sequences...were used to corrected
l. 113 articles "the" or plural needed corrected
L. 129 change "at" to "in" corrected
l. 224 ia not a sentence corrected
l.226 number disagreement Primeres...doesn't corrected
also in line 259 corrected
l.238 "was ground" corrected
l.273 "data are shown" corrected
l. 287 corrected
l. 298-99 corrected
l.326 delete "an" corrected
